# On children’s motives to influence parents’ long-term care insurance purchase: evidence from Switzerland

**DOI:** 10.1057/s41288-022-00273-7

**Published:** 2022-05-03

**Authors:** Christophe Courbage, Guillem Montoliu-Montes, Joël Wagner

**Affiliations:** 1 Geneva School of Business Administration, University of Applied Sciences Western Switzerland (HES-SO), Geneva, Switzerland; 2 Department of Actuarial Science, Faculty of Business and Economics (HEC), University of Lausanne, Lausanne, Switzerland; 3 Swiss Finance Institute, University of Lausanne, Lausanne, Switzerland

**Keywords:** Long-term care insurance, Adult children, Informal care, Bequest

## Abstract

Long-term care (LTC) is not only a concern for elderly individuals but also for their adult children, as the latter often provide financial support and informal care to their elderly dependents. Adult children may therefore have strong incentives to have their parents purchase LTC insurance. Using data from a 2019 Swiss survey, this article first identifies a set of variables, including self-reported interest about LTC insurance, whether elderly parents live with their children and if the latter have provided informal help with personal care, which help predict the interest of adult children in having their parents covered against LTC risk. Second, it investigates the main characteristics of children’s motives for influencing their parents to purchase LTC insurance, which are classified as either altruistic, i.e. related to parental well-being, or self-interested, i.e. related to the child’s well-being. The results offer valuable insights for both policymakers and insurers when designing public LTC policies and LTC insurance products.

## Introduction

The ageing of populations in most industrialised countries is accompanied by an increase in the need for long-term care (LTC), i.e. care for people dependent on help with their daily living activities. LTC is not only a concern for elderly individuals but also for their adult children (Courbage and Eeckhoudt 2012), as most of them provide financial support and informal care to their elderly dependents (Van Houtven et al. 2019). Adult children may therefore have strong incentives, whether altruistic or self-interested, to have their parents purchase LTC insurance. This is especially the case as LTC insurance entails obvious spillover effects on families, contrary to most insurance models for which the insurance beneficiary and purchaser are the same. The aim of this paper is to study children’s motives to influence parental LTC insurance ownership.

Compensation for the help adult children provide to their elderly parents is one motivation for having their parents insured. Indeed, adult children are the main providers of informal care, which could be detrimental to their health (Bom et al. 2019) and employment participation (Moussa 2019), and incurs high opportunity costs (van den Berg et al. 2005). Additionally, children may pay themselves for their parents’ LTC expenditures, especially if they feel compelled to take care of their dependent relatives (Klimaviciute et al. 2017). They may also become legally obliged to financially support their parents if they have exhausted their resources to cover their LTC needs. This is especially the case in countries such as Switzerland, Germany, France and Belgium, where the respective civil codes explicitly force adult children to assist their parents when in need (Sayn 2008). Hence, having parents purchase LTC insurance that covers the cost of formal care might relieve children of their informal care duties and allow them to avoid tapping into personal wealth to finance the possible LTC needs of their parents. In addition, LTC insurance makes it possible to protect children’s future inheritance from the cost of LTC^1^ (Pauly 1990; Courbage and Roudaut 2008).

While these motives are rather self-interested, adult children may also be attentive to their parents’ LTC coverage for altruistic reasons, in other words, simply because they are concerned about their elderly parents’ well-being (Becker 1974; Andreoni 1990).^2^ For instance, Hanewald et al. (2020) show that the main reason adult children in China would recommend a reverse mortgage to their elderly parents is to finance complementary care services and medical treatments. In the same spirit, adult children may see insurance coverage as bringing useful and complementary services to their parents (Dong et al. 2019). Alternatively, children may also want to avoid financial distress to their parents in the event of needing LTC or may be worried about their parents’ comfort during later life (Hanewald et al. 2020).

In this article, we investigate both the determinants and self-reported motives of adult children’s willingness to influence their elderly parents to purchase LTC insurance in Switzerland. To that aim, we use data from a novel survey conducted in 2019 on a sample of middle-aged individuals (40 to 65 years old). The survey first explicitly asked respondents whether they would be willing to encourage their parents or in-laws to buy LTC insurance. Second, as LTC insurance can serve multiple purposes to children, those respondents that were willing to influence their elderly parents to purchase LTC insurance were asked about their reasons, including concerns about their parents’ well-being (Hanewald et al. 2020), the burden of informal care (Bom et al. 2019; Moussa 2019), bequest motives (Pauly 1990) and legal responsibility (Sayn 2008).

We are aware of few papers looking at the role of adult children in their elderly parents’ LTC financing decisions. Cohen et al. (2000) conducted a survey stressing that primary informal caregivers play an important role in the purchase of LTC insurance by their elderly relatives in the U.S. Related to this, Zhou-Richter et al. (2010) use a survey in Germany, showing that the more adult children are informed about LTC risk, the more likely private LTC insurance is purchased, either by the adult children themselves on behalf of their parents or by the parents under the influence of their adult children. Sperber et al. (2014) carried out a survey in the U.S. showing that adult children successfully influence their parents to purchase LTC insurance by framing insurance with respect to their values concerning autonomy for themselves and their children. Recently, Hanewald et al. (2020) studied the interest among 45–69-year-old urban Chinese and their adult children in reverse mortgages as a way to finance the retirement income of the former. They show that a very large rate of children would recommend this product to their parents if it is described in an easy-to-understand way and directly addresses their key concerns. On the theoretical side, Courbage and Eeckhoudt (2012) look at both the optimal levels of insurance and informal care chosen by the child to protect a parent against LTC risks. In particular, they consider two scenarios regarding the child’s motives for having their parents protected against LTC risks. They first consider the case where the child is only interested about his or her wealth and the wealth of the parent. They also consider the case where the child is altruistic and derives satisfaction from the well-being of the parent. They show that, in the presence of child altruism, LTC insurance stimulates the supply of informal care.

The main contribution of our article is twofold. First, it identifies a set of variables, including socio-economic factors, family characteristics and parental LTC needs, which help predict the interest of adult children in having their parents covered against LTC risk. In this respect, our article is of an exploratory nature and all potential explanatory variables are equally important *ex ante*. Second, it investigates the main characteristics of children’s motives for influencing their parents to purchase LTC insurance, classifying these as either self-interested or as altruistic in the spirit of Courbage and Eeckhoudt (2012). To the best of our knowledge, no empirical study on this topic exists for Europe, with the exception of Germany (Zhou-Richter et al. 2010). However, while Zhou-Richter et al. (2010) focus on the role of children’s information about LTC risk, our article points to multiple channels through which adult children could influence their parents’ demand for LTC insurance. For the first time, it investigates empirically the determinants of children’s motivations to influence their parents’ decision to purchase LTC insurance.

We show that individuals with self-reported interest in LTC insurance, who live with their parents and have provided informal help with personal care are more likely to influence their parents to purchase LTC insurance. Quantitatively, the marginal effect of reporting interest in LTC insurance is the largest amongst all explanatory variables. Indeed, those individuals reporting only little interest in LTC insurance are 16% more likely to influence their parents than those reporting no interest. As for the motives, we find that they can be classified either as ‘altruistic’, i.e. related to parental well-being, or as ‘self-interested’, i.e. related to the child’s well-being. We find that most respondents are likely to influence their parents for altruistic motives. We also find that respondents with less wealthy parents tend to influence their parents mainly for altruistic reasons, i.e. to avoid their economic ruin. Individuals with wealthy parents, or those who expect to pay large out-of-pocket LTC costs in case of dependency or whose own wealth is large, on the other hand, are more likely to influence their parents for self-interested motives, i.e. to protect their bequest, to avoid providing informal care or to avoid legal responsibilities towards parents in need.

Our results can be valuable both for policymakers and insurers, as knowing the profile of children who are willing to influence their parents’ LTC coverage and their motivations for doing so might be useful for the specific design of public LTC policies and LTC insurance products. In addition, this study can provide further insights for developing LTC insurance in Switzerland (Fuino et al. 2020), a country where such a market could be particularly attractive.

This article is structured as follows. In the next section, we briefly describe how LTC is financed in Switzerland. In the third section, we present the dataset and variables used. The fourth section empirically addresses the determinants of adult children’s willingness to influence their parents LTC insurance purchase, while the fifth section studies their motives. Concluding remarks are provided in the final section.

## LTC financing in Switzerland

Switzerland is a federal state with three levels of government: federal, cantonal (26 cantons) and municipal (about 2600 municipalities). It counts about 8 million inhabitants with four official languages: German (spoken by 62% of the population), French (23%), Italian (8%) and Romansh (0.5%).

The financing of LTC is decentralised (Weaver 2012). At the national level, health insurance funds, as part of compulsory social insurance, finance ambulatory LTC if it is related to sickness but not to old age. Other services, such as household assistance, activity therapy or food and board in nursing homes, are paid out-of-pocket by households (Gentili et al. 2017). Individuals who cannot cover these expenses from their own assets or retirement income can apply for supplementary payments from the national public old-age (AHV) and invalidity (IV) insurance schemes or for social assistance from municipal governments. Hence, health insurance, cantons and municipalities finance approximatively 60% of LTC costs. The remaining 40% is covered by households (European Commission 2018).

Supplemental private insurance plays a minor role in LTC financing. The LTC insurance market devoted solely to cover the risk of LTC is small or even inexistent in Switzerland. This is rather paradoxical given the high development of other markets like health insurance. Private LTC coverage is provided under life insurance or supplementary health insurance. Concerning life insurance, products involve paying a life annuity to people reaching the age of 65 and who need daily help. As for complementary health insurance, different models exist. In some cases, insureds can choose an indemnity ceiling of a certain amount per day, from which home care costs or food and board costs in nursing homes will be reimbursed. Other providers offer partial LTC coverage as part of supplemental health insurance covering a wider range of healthcare services. Finally, some private health plans partially insure disability by providing daily lump sums in case of home care use or nursing home stays (Weaver 2012).

Switzerland is a country where private LTC insurance could be particularly attractive, given the rapid ageing of its population, the large private health insurance market and the high level of out-of-pocket LTC expenses. This context calls for better understanding the channels and drivers of LTC insurance purchase decisions, including the influence of children.

## Data and variables

### Survey questions, available data and dependent variable

The survey used in this work relies on a specific questionnaire covering several topics related to LTC financing. The target population covers residents of the German and French linguistic regions of Switzerland^3^ aged between 40 and 65 years. The core of the survey is composed of four parts dealing with respondents’ family background, informal care supply, perception of LTC risks and preferences toward LTC financing. It also includes questions related to the respondents’ attitudes toward risk and the future in general, socio-demographic characteristics and professional and economic situation.

The data collection process was conducted online in February 2019 by a professional polling agency and resulted in a representative, i.e. randomly selected, sample of 1066 individuals. Special attention was paid to have sufficient respondents with dependent parents and providing informal care. Therefore, a stratified sampling on three stratum was performed, according to the following proportions. One third of respondents were individuals with dependent parents and providing informal care; one third were individuals with dependent parents but not providing informal care; and the remaining third corresponded to individuals with any dependent relative. Within each stratus, the sample was additionally stratified by gender (50% men and 50% women), age group (40% aged 40–49, 40% aged 50–59 and 20% aged 60–65) and linguistic region (67% German and 33% French). The sample weights of the second stratification were approximately equal to the population weights, with the exception of the French-speaking linguistic region, which was over-represented.

Given the nature of our research question, we restrict our final sample to those respondents having at least one parent or parent-in-law alive. This leaves us with a final dataset containing 881 observations.

The main dependent variable aims to capture the willingness of children to influence their parents’ or in-laws’ coverage against LTC risk by coding the answer to the following question:

*Have you tried to influence or are you willing to influence your parents or parents-in-law to contract LTC insurance?*

The answer to this question is binary and respondents could choose among the options ‘Yes’ and ‘No’. This question was asked at the end of the survey, when the definition of LTC insurance,^4^ the different concepts of LTC financing and the average amount of out-of-pocket LTC expenditure in Switzerland had already been presented to respondents. Respondents who answered affirmatively to the previous question were additionally asked about their motives. They include:

- I would like to avoid my parents’ (-in-law) economic ruin.
- My parents’ (-in-law) savings are not enough to pay for their LTC expenses.
- I could avoid the burden of providing care to my parents (-in-law).
- I will protect my future bequest, by avoiding my parents (-in-law) having to pay for formal care.
- I am legally responsible to help my parents (-in-law) if they do not have enough means.

This set of motives addresses, respectively, concerns about parents’ financial well-being (Hanewald et al. 2020), avoiding the burden of informal care (Schulz and Beach 1999; Moussa 2019), bequest motives (Pauly 1990) and legal responsibility (Sayn 2008). Answers are constructed as a five-point Likert-type scale, with item 1 being equal to *Totally disagree* and item 5 being equal to *Completely agree*. The first two motives can be qualified as ‘altruistic’ since they reveal that adult children want to influence their parents (-in-law) to improve their welfare. The last three motives can be referred to as ‘self-interested’ since they reveal respondents who want to influence their parents (-in-law) to improve their own well-being. Naturally, individuals’ preferences in practice could include a combination of both altruistic and self-interested motives (Andreoni 1990).

### Independent variables

We classify the potential determinants of adult children’s willingness to influence their parents-in-laws’ LTC insurance purchase by considering respondents’ socio-economic situation, family composition, parental LTC needs and individual preferences, as well as some other classical control variables.

We start by considering various socio-economic factors, including the respondent’s working status, the highest level of education, income, main residence ownership (as a proxy of personal wealth) and parental level of wealth. This last variable is defined as the maximum wealth between the respondent’s parents’ and parents-in-laws’ wealth. Income, working status and education, as proxies for opportunity cost of informal care (Moussa 2019), should be positively related to the willingness to influence.

We also consider several variables describing the main characteristics of respondents’ family structure. They include marital status, whether the respondent has at least one brother or sister, the number of individuals residing in the respondent’s household, the frequency of the respondent’s contacts with siblings and the number of children younger than 18 years living in the respondent’s household. Influencing parental LTC coverage might be closely related to the degree of concern for parents’ financial well-being (Hanewald et al. 2020), the strength of family ties (Costa-Font 2010) or the presence of young children in the household leaving less time to care for elderly parents.

Having a dependent parent as well as providing informal care are also included as explanatory variables. Further, we look at the level of dependency of the respondent’s parents, the nature of informal care provided (e.g. ADL, IADL, administrative activities), the respondent’s self-reported degree of physical and psychological burden when providing informal care and the pathologies faced by dependent parents, if any (e.g. mental disease, neurological pathology).

Additional variables related to the respondent’s preferences and perception of LTC financial risks are considered. They include self-reported interest in LTC insurance, whether the respondent wants to be cared for by the family in case of dependency (as a proxy of the individual’s preference for informal care) and expectations about out-of-pocket LTC payments in case of dependency. We also examine the respondent’s self-reported understanding about LTC insurance, given that financial literacy has been shown to be a strong predictor of demand for financial services (Lusardi and Mitchell 2011; Cole et al. 2011).

Finally, the usual controls such as gender, age, nationality and self-reported health are also included. Detailed information about the variables considered as potential covariates and their brief description is reported in Table 1.

**Table 1:** Summary of variables used and survey questions

	Variable	Question in the survey	Answers/built categories
	*Dependent variable (WI)*		
1	Willingness to influence	Have you tried or are you willing to influence your parents or in-laws to subscribe to LTC insurance?	Yes, no
	*Socio-economic factors (SOC)*		
2	Working status	What is your current profession?	Employed, retired, other
3	Education	What is your highest level of education?	Mandatory, high school, higher education
4	Income	What is your monthly net income?	≤ 3000, 3001–5000, 5001–7000, 7001–9000, > 9000, NA
5	Housing	Concerning your main residence, are you…	Tenant, owner, other
6	Parental wealth	How do you estimate your parents’ net wealth? And your in-laws’ net-wealth?	Very low, low, high, very high
	*Family characteristics (FC)*		
7	Household members	How many people are there in your household including you?	1, 2, 3, 4 or more
8	Siblings	Do you have any brother or any sister?	Yes, no
9	Married	What is your civil status?	Married, not married
10	# of coresident children	How many children younger than 18 years are there in your household?	0, 1, 2 or more
11	Contact with siblings	During the last 12 months, how often have you contacted your siblings? Think only about the person you contacted the most often if you have several siblings	Never, less than every 2 weeks, every 2 weeks, weekly, several times a week, daily
	*Parent LTC needs (LTC)*		
12	LTC needs	During the last 12 months, did any of your parents / in-laws have any difficulties carrying out independently a daily living activity (take a bath or a shower, go to the toilet, to get dressed…)?	Yes, no
13	Intensity of dependency	With how many daily living activities does your father / mother (-in-law) have functional limitations?	0, 1, 2, 3, 4 or more
14	Activity	With which activities (ADL, IADL, administrative tasks or emotional support) have you helped your father / mother (-in-law) during the last twelve months?	Matrix with 4 variables. Yes, no
15	Informal care burden	Self-reported burden of informal care, built from an index composed of 7 questions	Scale. 1–15
16	Pathology	Do / did any of your parents or in-laws suffer from any of the following diseases: a mental, muscoskeletal, cardiovascular or neurological problem or cancer?	Matrix with 5 variables. Yes, no
	*Preferences (PRE)*		
17	Interest in LTCI	Are you interested in purchasing a LTC insurance?	Not at all, little interest, some interest, strong interest
18	Out-of-pocket LTC costs	If you became dependent, how much do you think you will have to pay out-of-pocket for LTC?	Nothing, little part, important part, almost all, don’t know
19	Help with ADL by family	If you became dependent, would you like receive personal care from relatives, neighbours or friends?	Yes, no
20	Understanding LTCI	Please, indicate your degree of understanding concerning the financial protection provided by LTC insurance	Not clear at all, unclear, fairly clear, very clear
21	Planning	In general, are you interested on planning the future?	Scale. 1–10
22	Risk aversion	In general, are you a person willing to take risks?	Scale. 1–10
	*Control variables (CV)*		
23	Language	Language of the questionnaire	German, French
24	Gender	You are a…	Male, female
25	Swiss	Which is your nationality? In case of double-nationality, please indicate your nationality at birth	Non-Swiss, Swiss
26	Age	How old are you?	40–45, 46–50, 51–55, 56–60, 61–65
27	Health	How do you perceive your own health status in general?	Very bad, bad, fair, good, very good

### Descriptive statistics

Tables 2 and 3 provide some descriptive statistics of the dependent variable.

**Table 2:** Percentage of respondents willing to influence their parents/in-laws to purchase LTC insurance

Willingness to influence	*N*	% of *N*
Yes	239	27.13
No	642	72.87
Total	881	100.00

**Table 3:** Motives of the respondents willing to influence their parents (-in-law) to purchase LTC insurance (*N* = 239)

Motives to influence	Strongly agree (%)	Agree (%)	Neutral (%)	Disagree (%)	Totally disagree (%)
Avoid parents’(-in-law) ruin	52.72	24.69	14.23	4.60	3.77
Insufficient parental savings	41.00	23.85	22.18	10.46	2.51
Avoid providing help	17.15	21.34	30.96	14.64	15.90
Bequest motive	15.48	20.50	28.03	12.97	23.01
Legal responsibility	17.57	20.92	24.27	16.74	20.50

Two hundred and thirty-nine individuals, representing 27% of all respondents, replied that they tried to influence or are willing to influence their parents (-in-law) to subscribe to LTC insurance. When it comes to the self-reported motives of surveyed individuals, the two ‘altruistic’ ones, i.e. avoiding parents’ economic ruin and insufficient savings, had the strongest support. Indeed, 77% and 65% of respondents that were willing to influence their parents answered *Agree* or *Strongly agree* with the first and second motive, respectively. Much less support is found for the ‘self-interested’ motives, i.e. to avoid the burden of helping them, to protect bequest or because children are legally responsible for their parents in case of necessity. The rate of agreement (*Agree* or *Strongly agree*) for each of these motives is around 37%.

Table 4 provides the sample mean of the potential determinants of the dependent variable as well as the share of respondents willing to influence their parents to purchase LTC insurance in each category of the explanatory variables.

**Table 4:** Sample mean and share of respondents willing to influence in each category (in percent)

	*Mean | Willing*		*Mean | Willing*		*Mean | Willing*
*Socio-economic factors*					
Working status		Income		Housing	
Employed	78.09 | 28.05	3000 or less	11.80 | 23.08	Tenant	65.38 | 25.35
Retired	6.47 | 24.56	3001–5000	23.04 | 25.12	Owner	33.37 | 30.61
Other	15.44 | 23.53	5001–7000	19.86 | 26.86	Other	1.25 | 27.27
Education		7001–9000	13.28 | 26.50	Parental wealth	
Mandatory	6.02 | 30.19	More than 9000	19.39 | 40.68	Very low	19.98 | 24.43
High school	57.66 | 23.62	NA	18.62 | 23.17	Low	49.72 | 27.17
Higher education	36.32 | 32.19			High	28.38 | 27.60
				Very high	1.92 | 47.06
*Family characteristics*					
Household members		Married		Contact with siblings	
1	21.68 | 17.80	No	38.93 | 23.03	Never	17.59 | 29.03
2	34.17 | 25.91	Yes	31.07 | 29.74	Less every 2 weeks	32.35 | 22.81
3	17.93 | 29.11	# coresident children		Every 2 weeks	14.07 | 18.55
4 or more	26.22 | 35.06	0	66.89 | 22.58	Weekly	15.89 | 30.00
Siblings		1	15.21 | 33.58	Several times a week	14.64 | 32.56
No	12.15 | 28.04	2 or more	17.93 | 38.61	Daily	5.45 | 45.83
Yes	87.85 | 27.00				
*Parent LTC needs*					
LTC needs		Help ADL		Mental pathology	
No	35.07 | 22.33	No	79.23 | 23.50	No	86.27 | 26.05
Yes	64.92 | 29.72	Yes	20.77 | 40.98	Yes	13.73 | 33.88
Intensity of dependency		Help IADL		Musco-skeletal pathology	
No dependent	35.07 | 22.33	No	69.35 | 24.71	No	64.36 | 25.93
1	15.55 | 24.82	Yes	30.65 | 32.57	Yes	35.64 | 29.30
2	14.42 | 25.98	Help admin		Cardiovascular pathology	
3	12.71 | 32.14	No	74.01 | 25.15	No	77.30 | 24.23
4 or more	22.50 | 34.18	Yes	25.99 | 32.75	Yes	22.70 | 37.00
		Help company		Neurological pathology	
		No	70.15 | 25.08	No	92.85 | 26.53
		Yes	29.85 | 31.94	Yes	7.15 | 34.92
		Informal care burden		Cancer	
		No	58.91 | 33.15	No	95.91 | 27.22
		Linear	3.471 |−	Yes	4.09 | 25.00
*Preferences*					
Out-of-pocket LTC costs		Interest in LTCI		Understanding LTCI	
Don’t know	12.49 | 26.36	Not at all	14.98 | 5.30	Not clear	18.27 | 14.91
Nothing	20.89 | 20.65	Little	43.59 | 21.09	Unclear	40.52 | 25.77
Little part	25.20 | 27.93	Some	34.62 | 40.00	Fairly clear	30.53 | 33.46
Important part	25.43 | 29.02	Strong	6.87 | 48.33	Very clear	10.67 | 35.11
Almost all	16.00 | 31.91	Help ADL family		Planning	7.518 |−
		No	48.35 | 23.94	Risk aversion	5.645 |−
		Yes	61.65 | 30.11		
*Control variables*					
Language		Age		Health	
German	66.97 | 27.63	40–45	30.87 | 33.46	Very bad	1.48 | 23.08
French	33.03 | 26.12	46–50	22.02 | 26.80	Bad	11.46 | 23.76
Gender		51–55	22.13 | 21.54	Fair	32.12 | 32.86
Male	49.94 | 26.57	56–60	14.42 | 25.20	Good	40.30 | 25.07
Female	50.06 | 27.66	61–65	10.56 | 23.66	Very good	14.64 | 23.26
Swiss					
Not Swiss	19.30 | 37.65				
Swiss	80.70 | 24.61				
*N*	881				

Most respondents are employed, live in rented accommodation and have a high school education. Additionally, most individuals nominate their parental wealth as *Low* or *Very low*. The monthly income distribution is relatively uniform with the modal class at CHF 3001–5000.^5^ Regarding the family characteristics, 56% of respondents live in a household with one or two individuals, approximately 60% are married and one third coresides with children under the age of 18. Very few respondents have regular contact with their siblings. Concerning the variables related to the respondents’ parents’ LTC needs, 42% of those surveyed declared that they provide some form of informal help, among which 20% provide help with ADL (personal care) and 31% with IADL (practical household help). Forty-one percent of respondents (almost all of those who provide care) declared that they suffer from some burden related to the provision of informal help. Concerning individual preferences, most of those interviewed report being aware that they will face some out-of-pocket expenditures in case of dependency. They mainly show little or some interest in LTC insurance. Finally, 80% of our sample is Swiss and only 13% of respondents declared that they were in bad or very bad health.

Table 4 provides a first approximation of the profile of individuals willing to influence their parents (in-law) to purchase LTC insurance. Indeed, respondents earning a monthly income greater than CHF 9000, showing some or strong interest in LTC insurance, or providing help with ADL have a relatively high probability of being willing to influence their parents (i.e. greater than 40%).

In the next sections, we first investigate the determinants of the respondents’ willingness to influence their parents or in-laws to purchase LTC insurance. Second, we study the different motives for influencing parents’ or in-laws’ LTC coverage.

## Determinants of the willingness to influence parents’ LTC insurance coverage

We first aim to shed light on the direction and magnitude of the relationship between the set of potential independent variables considered and the main dependent variable.

### Econometric specification

In this subsection, we perform a series of probit regressions obtained from the following model:

1 $$WI_{i} = \alpha_{j} + \beta_{1}^{j} SOC_{j,i} + \beta_{2}^{j} FC_{j,i} + \beta_{3}^{j} LTC_{j,i} + \beta_{4}^{j} PRE_{j,i} + \beta_{5}^{j} CV_{j,i} + \varepsilon_{j,i}$$

where *j* indicates each multivariate regression estimated and *i* indicates individual observations. $WI_{i}$ is a binary variable quantifying respondent *i*’s willingness to influence their parents (-in-law) to subscribe to LTC insurance. The use of a probit specification is justified by the binary nature of $WI_{i}$.

$SOC_{j,i}$ refers to the set of socio-economic factors in Table 1 selected as independent variables in the regression model *j*. Similarly, $FC_{j,i}$ encompasses the variables related to family composition, $LTC_{j,i}$ those related to the respondent’s parents’ LTC needs and $PRE_{j,i}$ those linked to the respondent’s preferences. Finally, $CV_{j,i}$ includes the control variables selected for the model *j* and $\varepsilon_{j,i}$ is a set of *i.i.d.* random variables following a standard normal distribution. As previously stressed, and given the exploratory nature of our work, all potential explanatory variables are considered equally important *ex ante*.

The main core of independent variables included in each specific model is selected from the optimisation of the Bayes (BIC) and the Akaike information criteria (AIC). More specifically, a variable is included in our model only if it decreases the value of the selected criterion. This method ensures that any given selected variable improves the model’s goodness-of-fit without substantially raising the risk of overfitting.^6^

It should be stressed that the binary variables *LTC needs* and *Language* have been included in all regressions *ex-post* (i.e. once the models have been estimated), regardless of the information criteria. This is done to control for the fact that the prevalence in the sample of individuals with and without dependent parents and from the French-speaking linguistic reason is not representative (see “Survey questions, available data and dependent variable” section). As stressed earlier, the sampling design required a specific proportion of individuals with dependent parents to ensure a large enough number of respondents in this segment. We additionally performed variance inflation factor (VIF) checks on all regressions. No high values were found for these tests, confirming the absence of multicollinearity issues in our results.

It should also be noted that the econometric specification depicted in Eq. (1) only measures association and not causality between the dependent and independent variables. A causal interpretation for the control variables’ marginal effects is beyond the scope of this paper.

Finally, we did not include interactions in our specification because the optimisation of such models had difficulties to converge towards a stable result.

### Empirical results

The numerical results of the different multivariate models calibrated from Eq. (1) are presented in Table 5. Since the interpretation of probit models’ coefficients is quite complex, we report average marginal effects (AME), i.e. the mean of all individuals’ marginal effects for each variable or category, the interpretation of which is more intuitive.

**Table 5:** Multivariate probit models (average marginal effects)

Dependent variable: willingness to influence	Model 1 (BIC)	Model 2 (AIC)	Model 3 (AIC alternative)	Model 4 (‘Healthy’ parents)	Model 5 (No preference)
*Income (ref: Less than 9000)*					
More than 9000	−	−	0.106** (0.043)	0.106* (0.061)	0.145*** (0.047)
NA	−	−	0.015 (0.037)	0.049 (0.051)	− 0.014 (0.037)
*# of coresident children (ref: 0)*					
1	0.083** (0.040)	0.087** (0.040)	0.087** (0.040)	0.045 (0.057)	0.093** (0.043)
2 or more	0.162*** (0.039)	0.164*** (0.039)	0.157*** (0.039)	0.140*** (0.054)	0.146*** (0.041)
LTC needs (ref: no)	− 0.003 (0.035)	− 0.024 (0.037)	− 0.029 (0.037)	− 0.023 (0.052)	− 0.036 (0.039)
Help ADL (ref: no)	0.122*** (0.040)	0.111** (0.048)	0.108** (0.048)	0.168 (0.173)	0.123** (0.051)
Help IADL (ref: no)	−	− 0.106*** (0.039)	− 0.112*** (0.039)	− 0.144** (0.059)	− 0.086** (0.042)
Informal care burden (linear)	−	0.017*** (0.006)	0.018*** (0.006)	0.030** (0.014)	0.018*** (0.006)
Cardiovascular (ref: no)	0.093** (0.038)	0.100*** (0.038)	0.099*** (0.037)	0.068 (0.083)	0.088** (0.040)
*Interest in LTCI (ref: no)*					
Little	0.157*** (0.028)	0.159*** (0.029)	0.158*** (0.029)	0.156*** (0.033)	−
Some	0.346*** (0.034)	0.329*** (0.034)	0.323*** (0.035)	0.336*** (0.042)	−
Strong	0.412*** (0.066)	0.382*** (0.068)	0.383*** (0.068)	0.367*** (0.093)	−
*LTCI understanding (ref: not clear)*					
Unclear	−	0.102*** (0.037)	0.106*** (0.037)	0.057 (0.051)	−
Fairly clear	−	0.133*** (0.039)	0.127*** (0.039)	0.088 (0.055)	−
Very clear	−	0.154*** (0.055)	0.154*** (0.054)	0.072 (0.072)	−
Language (ref: German)	− 0.038 (0.029)	− 0.052* (0.029)	− 0.187* (0.109)	− 0.092** (0.037)	− 0.030 (0.030)
Gender (ref: male)	−	0.045* (0.028)	0.054** (0.028)	0.023 (0.038)	0.041 (0.029)
Swiss (ref: non-Swiss)	−	− 0.085** (0.036)	− 0.086** (0.036)	− 0.039 (0.052)	− 0.112*** (0.039)
N	881	881	881	446	881
Pseudo *R*^2†^	10.90%	12.20%	12.45%	8.19%	4.62%
AIC	917.72	904.31	901.71	442.62	982.34
BIC	965.53	985.59	999.55	520.53	1044.49

Robust standard errors are reported in parentheses

The significance levels of the two-tailed hypothesis test are coded as follows: *Significance at 10% level, **Significance at 5% level, ***Significance at 1% level

^†^Mc. Fadden’s adjusted pseudo R

The model of the first column is the one minimising the BIC. It corresponds to the model with the smallest number of parameters, as this criterion penalises more strongly when additional variables with lower explanation power are included in the specification. Therefore, by construction, the model optimising the BIC displays the strongest determinants of the dependent variable.

Our first results indicate that showing self-reported interest in LTC insurance, having coresident children (especially more than two) and providing informal care for ADL (personal care) are the main determinants of the willingness to influence parents or in-laws to contract LTC insurance. Having a parent suffering from a cardiovascular disease is also a strong determinant of the dependent variable. The effect of self-reported interest about LTC insurance mirrors the results of Zhou-Richter et al. (2010), who show that parents strongly increase their demand for LTC insurance if their adult children had purchased it for themselves. Quantitatively, the marginal effect of this covariate is the largest amongst all independent variables. For instance, those individuals reporting only little interest about LTC insurance are, on average, 16% more likely to influence their parents than those showing no interest (AME = 0.157). Following Zhou-Richter et al. (2010)’s interpretation, self-interest about LTC insurance by adult children can be seen as a proxy for LTC risk awareness. Hence, those who are more aware about LTC risks are more likely to influence their parents to purchase LTC insurance. It also means that if one recognises the usefulness of LTC insurance for oneself it seems rather natural that one would find it useful for parents (-in-law). Having coresident children leaves less time and resources for taking care of elderly parents and thus provides further incentives to influence them to purchase LTC insurance. The size effect of this variable is also important. Indeed, individuals with two or more co-esident children are on average 16% more likely to influence their parents than those without any coresident child (AME = 0.162). Finally, providing informal help with personal care (ADL) adversely impacts the caregiver’s physical and psychological health (Roth et al. 2015; Musich et al. 2017). The quantitative effect of this factor is also rather strong, since those individuals who provided help with personal care are 12% more likely to influence their parents and in-laws to purchase LTC insurance (AME = 0.122). Hence, having parents purchase LTC insurance that covers the cost of formal care relieves children from their informal care burden. This would explain why providing informal help with personal care is a strong driver of the decision to influence parents to purchase LTC insurance.

The model of the second column corresponds to the one optimising the AIC. This model specification includes more variables than the previous one, as the penalty of the AIC on the number of parameters is lower. The main results mirror those of the model optimising the BIC, except for the effect of informal care provision, which is slightly different in this second specification. As before, helping parents (-in-law) with their ADL is positively and significantly associated with the dependent variable. Moreover, the self-reported burden of informal care provision is now included in the model and has a statistically significant positive effect. However, after controlling for these two variables, providing help with IADL (i.e. practical household help) has, surprisingly, a negative and significant effect on influencing parental LTC coverage. As providing informal care for ADL is more intense than for IADL, influencing LTC insurance purchase would not necessarily be done to replace informal care but rather to reduce the burden of intense and painful care provision. Our findings are consistent with Bonsang (2008), who finds that informal care decreases the use of formal domestic help, but complements paid personal care. Our results show that the higher the burden, the higher the probability of influencing parents. For instance, for a medium level of burden (i.e. 7 on a scale with values up to 15) the marginal effect resulting from the sum of *Help ADL*, *Help IADL* and *Informal care burden* is rather strong (i.e. equal to 0.124).^7^ This second specification also shows that adult children’s understanding of LTC insurance products, a proxy of their financial literacy, is an important determinant of the willingness to influence parents from both a qualitative and quantitative point of view. For instance, having a very clear understanding of LTC insurance products increases the probability of being willing to influence parents and in-laws by 15.4% (AME = 0.154). Additionally, being a woman has a weak positive effect on influencing parents, while being Swiss and living in the French-speaking region has a negative effect. The marginal effect of these variables has a less intuitive economic interpretation, since such socio-economic factors could be reduced to control variables.

Interestingly, neither the respondents’ nor their parents’ or parents-in-laws’ economic situations are associated with the dependent variable in the models in columns 1 and 2. Finally, the binary variable *LTC needs* is not significant at the usual confidence levels, regardless of the model (Table 5).

### Robustness checks

In order to test the robustness of the results reported in columns 1 and 2 of Table 5, we performed four checks.

In the first check, we estimated the models maximising the BIC and AIC (columns 1 and 2 of Table 5) using a logit instead of a probit multivariate regression. The results of the logit regressions, which are available upon request, are qualitatively and quantitatively similar, although the fit is slightly better in the probit models.

For the second check, we wanted to manually validate the main models provided through the optimisation of the BIC and AIC. The objective of this check is to ensure that no potential determinant is excluded from the optimal specifications. We first regressed the dependent variable on all independent variables of Table 1 individually, in a series of simple univariate regressions. In each univariate regression, we additionally tested the effect of its correspondent independent variable under different forms. For instance, if *Age* and *Health* are defined in Table 1 as categorical variables, we checked them as well under a linear form. We also allowed the variables *Income* and *Parental Wealth* to be binary, *Informal care burden* to be categorical, etc. Then, in a second step, we tested in the specifications of columns 1 and 2 of Table 5 the effect of those variables that were significant in the univariate regressions but were not included in the main models.^8^ The model of the third column of Table 5 corresponds to the specification maximising the AIC, consistent with the second check. The only change with respect to the main models is that the binary factor ‘Income > 9000’, corresponding roughly to the last decile of the Swiss net income distribution (FSO 2020b), is incorporated as a determinant and has a positive, significant effect. The size effect of this factor is rather strong. Indeed, individuals with a monthly income larger than CHF 9000 are on average 11% more likely to influence their parents or in-laws (AME = 0.106, see model 3 in Table 5). The other potential determinants tested that were significant in the univariate regressions were not significant in the multivariate model and provided a worse AIC. Hence, adult children with very high incomes are more likely to influence their parents’ and in-laws’ LTC insurance purchase decisions. This result can be explained by the fact that very high-income individuals have a higher opportunity cost of providing informal care or have more resources available to buy LTC insurance for their parents.

In the third check, we controlled for the potential ineligibility of elderly parents for LTC insurance. The model of the fourth column of Table 5 corresponds to the third robustness check. In this model, we ran the third column’s model on a subsample of respondents whose parents are not dependent or only need little help.^9^ This framing allowed us to make sure that respondents’ parents are eligible for LTC insurance, given that they are not yet dependent.^10^ Our main results do not change much in the last model, except that the magnitude and significance of the variable *LTCI understanding* are much lower. Self-reported interest about LTC insurance, having more than two coresident children and informal care provision are still the strongest determinants of the dependent variable. Moreover, the sign and magnitude of these variables’ coefficients are similar. However, the significance levels of the variable related to the number of coresident children and of the ones defining informal care provision are lower, since standard errors are much higher due to the reduced sample size.

Finally, in the fourth robustness check we wanted to investigate the effect of the variables related to respondents’ preferences (i.e. *Interest in LTCI* and *LTCI Understanding*, see Table 1) on the main results. Indeed, the way they are defined, these covariates are strongly related with the dependent variable, as the subjective choices they aim to measure are rather similar. This situation could have an impact on our results. To address this issue, in the model of the fifth column of Table 5 we estimate the third column’s model, removing the factors *Interest in LTCI* and *LTCI Understanding*. The average marginal effects of the rest of the covariates do not suffer major changes; however, the model’s Pseudo-R^2^ is substantially reduced from 12.45% to 4.62%, showing the large explanatory power of the omitted covariates.

## Motivation for influencing parents’ LTC coverage

We now focus on the respondents’ self-reported motives for influencing their parents to purchase LTC insurance. In particular, we study the relationship between the five motives for influencing LTC coverage presented previously and the profile of the respondents who tend to agree with the ‘altruistic’ versus the ‘self-interested’ motives.

The descriptive statistics (see Table 3) indicate that respondents largely agree with the first two motives, i.e. avoiding parents’ economic ruin and insufficient savings, while their degree of agreement is lower for motives three to five, i.e. avoiding having to provide informal care, the bequest motive and the legal responsibility motive.

To further study the relationship between the set of motives, we compute the covariance and correlation matrices of the respondents’ level of agreement on the different motives. The individuals’ level of agreement is quantified by numerically coding their answers from 1 to 5, with 1 corresponding to the lowest level of agreement (*Totally disagree*) and 5 to the highest (*Strongly agree*). Therefore, we assume that the level of agreement as defined by this measure is approximately continuous. The motives’ covariance and correlation matrices are displayed in Table 6.

**Table 6:** Covariance (left) and correlation matrix (right) of the different motives’ level of agreement

	Avoid ruin	Insuff. savings	Avoid help	Bequest	Legal resp.	Avoid ruin	Insuff. savings	Avoid help	Bequest	Legal resp.
Avoid parents’ (-in-law) ruin	1.165					1				
Insufficient parental savings	0.522	1.272				0.428	1			
Avoid providing help	0.227	− 0.021	1.681			0.162	− 0.014	1		
Bequest motive	0.118	0.073	0.830	1.877		0.080	0.047	0.468	1	
Legal responsibility	0.108	0.040	0.409	0.595	1.907	0.072	0.026	0.229	0.315	1

In general, the intensity of agreement across the different motives is positively correlated, with the exception of ‘Insufficient parental savings’ and ‘Avoid providing help’, the correlation for which is negative but very low. This observation indicates that, in general, respondents tend to agree (or disagree) on the five motives. From Table 6, we distinguish between two groups. On the one hand, we have the altruistic motives ‘Avoid parents’ ruin’ and ‘Insufficient parental savings’ with a correlation of 43%. On the other hand, the self-interested motives ‘Avoid providing help’, ‘Bequest motive’ and ‘Legal responsibility’ correlate at levels between 23 and 46%. The correlation between elements of the different groups is, instead, much lower.

In a second step, we perform a Principal Components Analysis (PCA) on the covariance matrix of Table 6. Our use of PCA is motivated by two objectives. First, it allows to further study the relationship between the five motives. Second, it allows to study the profile of respondents agreeing to a specific group of similar motives, either altruistic or self-interested. A summary of the PCA for the different motives is displayed in Table 7.

**Table 7:** Principal component analysis on the level of agreement for the different motives (eigenvectors on the columns)

Motives to influence	t_1_	t_2_	t_3_	t_4_	t_5_
Avoid parents’ (-in-law) ruin	0.2661	0.8439	− 0.0080	0.2441	− 0.5634
Insufficient parental savings	0.1295	0.9708	0.1107	− 0.2368	0.4892
Avoid providing help	0.9563	− 0.0560	− 0.5608	0.6186	0.2425
Bequest motive	1.1217	− 0.1230	− 0.3531	− 0.6697	− 0.1494
Legal responsibility	0.9123	− 0.1740	1.0086	0.1374	0.0244
Eigenvalues	3.09	1.70	1.47	0.97	0.64
% of variance	39.30	21.65	18.67	12.27	8.11
Cumulative % of variance	39.30	60.95	79.61	91.89	100

We focus on the first two dimensions of the PCA, which explain approximately 60% of the total variance. According to Table 7, their corresponding principal components are:

$$Z_{1} = 0.2661{ }Y_{1} + 0.1295 Y_{2} + 0.9563 Y_{3} + 1.1217{ }Y_{4} + 0.9123{ }Y_{5}$$

$$Z_{2} = 0.8439{ }Y_{1} + 0.9708{ }Y_{2} - 0.0560{ }Y_{3} - 0.1230{ }Y_{4} - 0.1740{ }Y_{5}$$

where $Y_{{\text{k}}} \in \left[ {Y_{1} , \ldots , Y_{5} } \right]$ corresponds to the degree of agreement on the kth motive. The first principal component $Z_{1}$ is the variable with the highest variance. As all coefficients are positive, $Z_{1}$ can be interpreted as the level of agreement on the five motives in general. An individual with a high (low) value of $Z_{1}$ will tend to agree (disagree) with the all five motives. The second component $Z_{2}$ has positive coefficients in the first two variables (*Avoid parents’ ruin* and *Insufficient parental savings*) and negative coefficients in the others (*Avoid providing help*, *Bequest motive* and *Legal responsibility*). The component $Z_{2}$ mirrors the two groups of motives identified previously, i.e. ‘altruistic’ and ‘self-interested’. Individuals with high $Z_{2}$ will tend to influence their parents’ insurance coverage thinking of the elderly’s interests while individuals with low $Z_{2}$ focus on their own interests.

The first principal component does not tell us much about the similarities and differences between the five motives. However, by studying the determinants of the second principal component $Z_{2}$, we unveil the profile of those respondents who are willing to influence their parents for ‘altruistic’ rather than ‘self-interested’ motives. To that aim, we regress the second principal component $Z_{2}$ on a set of covariates selected, as in the previous subsection, from the optimisation of the AIC after checking them under different forms. The results of this regression model are displayed in Table 8.

**Table 8:** Linear regression on the motivations of being willing to influence

Dependent variable: second principal component ($Z_{2}$)	
(Intercept)	− 0.233 (0.482)
*Working status (ref: active)*	
Retired	0.033 (0.330)
Other (incl. unemployed, homemaker…)	− 0.709*** (0.223)
Housing (ref: owner)	0.275* (0.159)
*Parental wealth (ref: very Low)*	
Low	− 0.575*** (0.219)
High	− 0.900*** (0.241)
Very high	− 1.506*** (0.475)
Help company (ref: no)	0.565*** (0.168)
Cardiovascular (ref: no)	− 0.413** (0.167)
Neurological (ref: no)	− 0.841*** (0.276)
*OOP LTC costs (ref: nothing or little part)*	
Important part or almost all	− 0.472*** (0.169)
Don’t know	− 0.046 (0.249)
Interest in LTCI	0.337*** (0.108)
Language (ref: German)	− 0.311* (0.164)
Health (ref: very bad or bad)	0.535** (0.239)
N	239
Adjusted *R*^2 †^	23.77%
AIC	758.18
BIC	813.80

Robust standard errors are reported in parentheses

The significance levels of the two-tailed hypothesis test are coded as follows: *Significance at 10% level, **Significance at 5% level, ***Significance at 1% level

The coefficient corresponding to ‘Other’ in the variable *Working Status*, which includes mainly unemployed people and homemakers, is negative and thus implies that this group would be more willing to influence their parents or in-laws to purchase LTC insurance for self-interested motives when compared to those who are retired or active. Respondents who expect to pay large out-of-pocket LTC costs in case of dependency also agree more with the self-interested motives. In addition, the variable *Housing* (with owner as a baseline reference) has a positive coefficient, while the effect of parental wealth is negative. This finding indicates that respondents whose parents’ or own wealth (proxied by residence ownership) are large also tend to be more willing to influence their parents’ or in-laws’ LTC coverage for self-interested reasons.

The effect of working status is driven by the fact that unemployed and homemakers assume the greatest responsibility if one of their parents becomes dependent. This observation is confirmed by the fact that these groups of respondents strongly agree with the legal responsibility motive. Our results also show that economic factors affect motives behind willingness to influence parental LTC coverage. In particular, the degree of agreement on altruistic versus self-interested motives to influence parental LTC coverage is strongly correlated with the respondent’s and parental wealth and expectations of out-of-pocket LTC costs. Whereas poor respondents (i.e. those who do not own their main residence) or those with less wealthy parents tend to influence their parents for altruistic reasons, i.e. to avoid their economic ruin, relatively rich individuals (i.e. those who own their main residence) report a lower degree of agreement for this set of motives. In particular, the distribution of motives within the subsamples of wealthier individuals or those with richer parents show that these types of respondents are much less in agreement with the ‘Insufficient parental savings’ motive. Finally, respondents that expect large out-of-pocket costs are more worried about their future bequest, which explains the negative effect of this variable on the principal component.

## Concluding remarks

In this paper, we explore the determinants of adult children’s willingness to influence their elderly parents’ LTC coverage in Switzerland and of their motives using data from a survey conducted in 2019.

Our results show that 27% of respondents are willing to influence their parents to contract LTC insurance. We find that reporting self-interest for LTC insurance, living with children under 18 years and providing informal care for ADL (personal care) are the strongest determinants of the willingness to influence parents’ or in-laws’ LTC insurance decisions. Quantitatively, the marginal effect of showing interest in LTC insurance is the largest amongst all explanatory variables. For instance, those individuals reporting only little interest in LTC insurance are 16% more likely to influence their parents than those showing no interest. Hence, those who are more aware of LTC risks (proxied by self-interest in LTC insurance) are more likely to influence the purchase of LTC insurance by their parents. In addition, personally recognising the usefulness of LTC insurance is also strongly related to influencing others to purchase it. Having children likely increases the opportunity cost of informal care as people with children have less time available to take care of their elderly parents. The quantitative effect of this variable is also important as, for example, respondents with two or more coresident children are 16% more likely to influence their parents than those without any coresident child. Providing informal help with personal care (ADL) is known to be time consuming and to adversely impact the physical and psychological health of informal caregivers (Roth et al. 2015; Musich et al. 2017). Hence, having parents purchase LTC insurance that covers the cost of formal care may relieve children of their most burdening informal care duties. Our results show that influencing parental LTC insurance purchase would not necessarily be done to substitute informal care with formal care, but rather to reduce the burden of intense and painful care provision. The higher the burden of providing informal care, the higher the probability of influencing parents or in-laws. For instance, for a medium level of burden, children are 12.4% more likely to influence their parents. We also find a positive effect of respondents’ understanding of LTC insurance on the dependent variable, showing the importance of adult children’s financial literacy in their willingness to influence parents’ LTC coverage. Finally, individuals with a high net income (i.e. greater than CHF 9000 per month) show a significantly higher willingness to influence their parents’ LTC insurance coverage. An explanation would be that adult children with large revenues have a high opportunity cost of providing informal care or that they can afford to pay for their parents’ LTC insurance premiums. The size effect of these variables is substantial, but smaller than that that of the variables previously mentioned. Having a very clear understanding of LTC insurance products increases the probability of willingness to influence parents and in-laws by 15.4%, while individuals with a monthly income larger than CHF 9000 are on average 11% more likely to influence parents or in-laws.

Regarding the motives for influencing parents’ or in-laws’ LTC coverage, we find that they can be grouped as either ‘altruistic’ or ‘self-interested’. Most respondents who are willing to influence their parents’ LTC coverage decisions do it for altruistic reasons, i.e. the interest of the elderly prevails over that of the child. Finally, we find that the motives for influencing parental LTC coverage have a socio-economic gradient, as individuals whose parents’ or own wealth (proxied by residence ownership) are large are more likely to influence their parents for self-interested motives. This is reflected by the coefficient corresponding to the variable *Housing* (with ‘owner’ as a baseline reference) having a positive sign in Table 8, while the coefficient corresponding to the variable *Parental wealth* has the opposite sign.

Our results offer various insights when it comes to managing LTC risks. A first is that the main reason children have for influencing their parents’ LTC insurance purchase is because they see it as a tool that would be beneficial to themselves. Nevertheless, this should be interpreted with caution since, as indicated in the “Robustness checks” section, the variables *Interest in LTCI* and *LTCI understanding* are strongly linked with the dependent variable and thus likely to be endogenous. Second, knowing the profile of those children who are willing to influence their parents’ LTC coverage and their motivations may be useful for the specific design of LTC financing policies. Indeed, our results indicate that one way to increase private LTC insurance among elderly parents is to directly target adult children with the relevant profiles (and whose parents are eligible), and to stress the various benefits for them of having their parents insured for LTC risks. It would be especially recommended, when targeting potential LTC insurance beneficiaries or policyholders, to focus on exogenous characteristics such as the number of children or the actual (or potential) burden of informal care provision. For instance, LTC insurance or benefits could be targeted to actual or potential informal caregivers or middle-aged individuals with children. In that respect, further research into casual effects would be required to make sure that interest in LTC insurance or understanding are characteristics worth targeting. Then, information on the product could be framed to children in such a way that it highlights how LTC insurance can avoid their parents’ financial distress during retirement or finance additional care costs. More generally, insurers should encourage the broader family to discuss the benefits of LTC insurance (Fuino et al. 2020). This may also create a spillover effect in which adult children could consider LTC insurance as an option for themselves, opening the path to contract LTC insurance at younger ages when the cost is lower and the premiums are more attractive.

Some limitations to this study need to be mentioned. First, as in the case of many survey-based studies, our work is observational in nature, meaning that estimates could be driven by omitted variables, although we have done our best to control for most variables. Second, the interpretation of the survey results and the conclusions must be taken with some circumspection since answers are self-reports and could be manipulated or affected by self-reporting bias A third limitation is that our data are cross-sectional and our sample relatively small, which, as previously stressed, deters us from performing a causal analysis, e.g. through a natural experiment. Therefore, our coefficients only reflect association, and a causal interpretation for the control variables’ marginal effects is beyond the scope of this paper. A fourth limitation is that the survey’s respondents expressed above all their willingness to influence insurance purchase, which may not necessarily reflect their real decision or lead to LTC insurance purchase by the parent. In that respect, to be successful in influencing parental purchase, Sperber et al. (2014) stressed the importance of framing LTC insurance in line with parents’ values concerning autonomy for themselves and their children. Finally, respondents are aged between 40 and 65 years old and their parents (-in-law) may be very old or already dependent, and therefore ineligible for LTC insurance or would face very high premiums. While we partially control for this issue, it could create a potential bias in survey answers should the respondent be aware of such information. However, these limitations should not seriously modify our results, which, we hope, contribute to better understanding the interests adult children take in how their parents’ LTC needs are covered.

## Footnotes

1 See Lambregts and and Schut (2020) for a comprehensive review of the factors driving LTC insurance uptake.

2 In Andreoni’s (1990) terms, this behaviour corresponds to perfect altruism, in contrast with imperfect altruism, for which economic agents do not care about others’ well-being but only about the act of giving.

3 The Italian-speaking region was not considered since it represents a small fraction of the Swiss population and Romansh speakers were included in the German linguistic region.

4 In the survey, LTC insurance is defined as “a complementary insurance that, against the payment of a premium, guarantees to the purchaser financial protection in the future if she/he has difficulties performing activities of daily living”.

5 In 2017, the average and median disposable income in Switzerland amounted to CHF 4554 and 4102, respectively (FSO 2020a).

6 The BIC is defined as $\ln \left( N \right)K - 2\log \hat{L}$, where *N* is the sample size, *K* the number of parameters of a given model and ${\text{log}}\hat{L}$ its log-likelihood. The AIC is defined as $2K - 2\log \hat{L}$. These criteria are minimised using the function ‘step’ of the R statistical software.

7 That is, 0.124 = 0.111 − 0.106 + 0.017 × 7 (see Table 5).

8 The auxiliary results of the second robustness check, that is, the univariate regressions and the multivariate models testing the effect of additional determinants, are available upon request.

9 We follow Klimaviciute et al. (2017) and consider a respondent to have ‘healthy’ parents if he/she declares having a parent with less than two limitations in activities of daily living. The threshold of two limitations is largely used to qualify for LTC insurance benefits and public support (Frank 2012; Courbage et al. 2020).

10 Another possibility to address this issue would be to consider a subsample of respondents below 50 years old, i.e. with relatively young parents. However, LTC insurance purchase at older ages is already possible through hybrid policies combining LTC and life insurance. Eligibility criteria based on ADL limitations, rather than on age, make more sense nowadays since hybrid products have almost substituted traditional LTC insurance in many countries. For instance, in the U.S. they represent 84% of market share, while traditional LTC insurance only accounts for 16% (AALTCI 2019).
